# Wavefront directionality and decremental stimuli synergistically improve identification of ventricular tachycardia substrate: insights from personalized computational heart models

**DOI:** 10.1093/europace/euac140

**Published:** 2022-08-25

**Authors:** Eric Sung, Adityo Prakosa, Stephen Kyranakis, Ronald D Berger, Jonathan Chrispin, Natalia A Trayanova

**Affiliations:** Department of Biomedical Engineering, Johns Hopkins University, Baltimore, MD 21287, USA; Alliance for Cardiovascular Diagnostic and Treatment Innovation, Johns Hopkins University, 3400 N. Charles Street, Baltimore, MD 21218, USA; Department of Biomedical Engineering, Johns Hopkins University, Baltimore, MD 21287, USA; Alliance for Cardiovascular Diagnostic and Treatment Innovation, Johns Hopkins University, 3400 N. Charles Street, Baltimore, MD 21218, USA; Department of Biomedical Engineering, Johns Hopkins University, Baltimore, MD 21287, USA; Alliance for Cardiovascular Diagnostic and Treatment Innovation, Johns Hopkins University, 3400 N. Charles Street, Baltimore, MD 21218, USA; Section of Cardiac Electrophysiology, Division of Cardiology, Department of Medicine, Johns Hopkins Hospital, Baltimore, MD 21287, USA; Alliance for Cardiovascular Diagnostic and Treatment Innovation, Johns Hopkins University, 3400 N. Charles Street, Baltimore, MD 21218, USA; Section of Cardiac Electrophysiology, Division of Cardiology, Department of Medicine, Johns Hopkins Hospital, Baltimore, MD 21287, USA; Department of Biomedical Engineering, Johns Hopkins University, Baltimore, MD 21287, USA; Alliance for Cardiovascular Diagnostic and Treatment Innovation, Johns Hopkins University, 3400 N. Charles Street, Baltimore, MD 21218, USA

**Keywords:** Ventricular tachycardia, Substrate electroanatomic mapping, Computer modelling, Imaging, Ablation

## Abstract

**Aims:**

Multiple wavefront pacing (MWP) and decremental pacing (DP) are two electroanatomic mapping (EAM) strategies that have emerged to better characterize the ventricular tachycardia (VT) substrate. The aim of this study was to assess how well MWP, DP, and their combination improve identification of electrophysiological abnormalities on EAM that reflect infarct remodelling and critical VT sites.

**Methods and results:**

Forty-eight personalized computational heart models were reconstructed using images from post-infarct patients undergoing VT ablation. Paced rhythms were simulated by delivering an initial (S1) and an extra-stimulus (S2) from one of 100 locations throughout each heart model. For each pacing, unipolar signals were computed along the myocardial surface to simulate substrate EAM. Six EAM features were extracted and compared with the infarct remodelling and critical VT sites. Concordance of S1 EAM features between different maps was lower in hearts with smaller amounts of remodelling. Incorporating S1 EAM features from multiple maps greatly improved the detection of remodelling, especially in hearts with less remodelling. Adding S2 EAM features from multiple maps decreased the number of maps required to achieve the same detection accuracy. S1 EAM features from multiple maps poorly identified critical VT sites. However, combining S1 and S2 EAM features from multiple maps paced near VT circuits greatly improved identification of critical VT sites.

**Conclusion:**

Electroanatomic mapping with MWP is more advantageous for characterization of substrate in hearts with less remodelling. During substrate EAM, MWP and DP should be combined and delivered from locations proximal to a suspected VT circuit to optimize identification of the critical VT site.

**What’s new**

- The substrate for ventricular tachycardia (VT) in hearts with less structural remodelling exhibits more variability to functional electroanatomic mapping (EAM) characterization than hearts with more structural remodelling.
- Electroanatomic mapping with multiple wavefront pacing (MWP) is more advantageous in characterizing the VT substrate in hearts with less structural remodelling.
- Decremental pacing (DP) can be synergistically combined with MWP to improve intraprocedural detection of remodelled substrate.
- Electroanatomic mapping with MWP only marginally improves the identification of critical VT sites.
- Decremental pacing from multiple locations near a suspected VT circuit greatly improves critical VT site identification.

## Introduction

Ventricular tachycardia (VT) is a life-threatening arrhythmia that affects patients with heart disease. Catheter ablation, a major adjunct to contemporary VT treatment regimens, is a minimally invasive procedure that aims to destroy disease-remodelled regions of the myocardium where VTs are most likely to manifest. Ablation is performed with electroanatomic mapping (EAM), spatiotemporal recordings of intracardiac electrical signals used to localize critical VT sites. However, identification of optimal VT ablation targets is non-trivial, and failure rates remain high, due in part to an unclear understanding of how EAM reflects underlying arrhythmogenic substrate.^1^

Substrate EAM, the characterization of electrophysiological abnormalities during sinus or paced rhythms, has emerged as the predominant mapping strategy as most VTs are haemodynamically unstable.^1^ Although various substrate-based ablation strategies have been proposed and tested, no single strategy has been shown to completely eliminate VT recurrences, and patients often have multiple procedures. This failure is in part explained by the fact that the VT substrate depends on the interplay between structural and functional properties^2^ and is unlikely to be fully characterized during sinus or pacing from a single location.

Two major strategies have emerged to overcome the limitations of traditional substrate mapping approaches: (i) multiple wavefront pacing (MWP),^3–5^ which maps wavefronts propagating in different directions, and (ii) decremental pacing (DP),^6–9^ which utilizes an early extra-stimulus. These functional mapping strategies better capture the dynamic properties of the VT substrate, and thus could improve identification of critical VT sites. However, it has not been systematically quantified how well MWP, DP, and even potentially the combination of both, uncover electrophysiological abnormalities on EAM that reflect underlying disease-induced remodelling and critical VT sites. Furthermore, prior studies have evaluated the utility of MWP and DP-based EAM strategies only using pacing locations independent of the patient-specific structural remodelling distribution.

The goal of this study is to investigate how MWP, DP, and the combination of both improves identification of electrophysiological abnormalities on EAM that reflect the patient-specific disease-induced remodelling and critical VT sites. Due to the intractability of clinically performing large numbers of paced maps for a patient, we develop a novel, personalized computational framework to perform substrate EAM in a virtual cohort of patient-specific 3D image-based, post-infarct heart models.^10^ In these heart models, we exhaustively pace from throughout the ventricle, creating high-resolution substrate maps from each pacing site. We compute multiple clinically relevant EAM features using an automated approach and quantify how well these EAM features reflect underlying infarct remodelling and critical sites for VT. Our results provide guidance on how best to apply functional mapping strategies to optimize identification of VT ablation targets.

## Methods

### Study workflow

**Figure 1:**
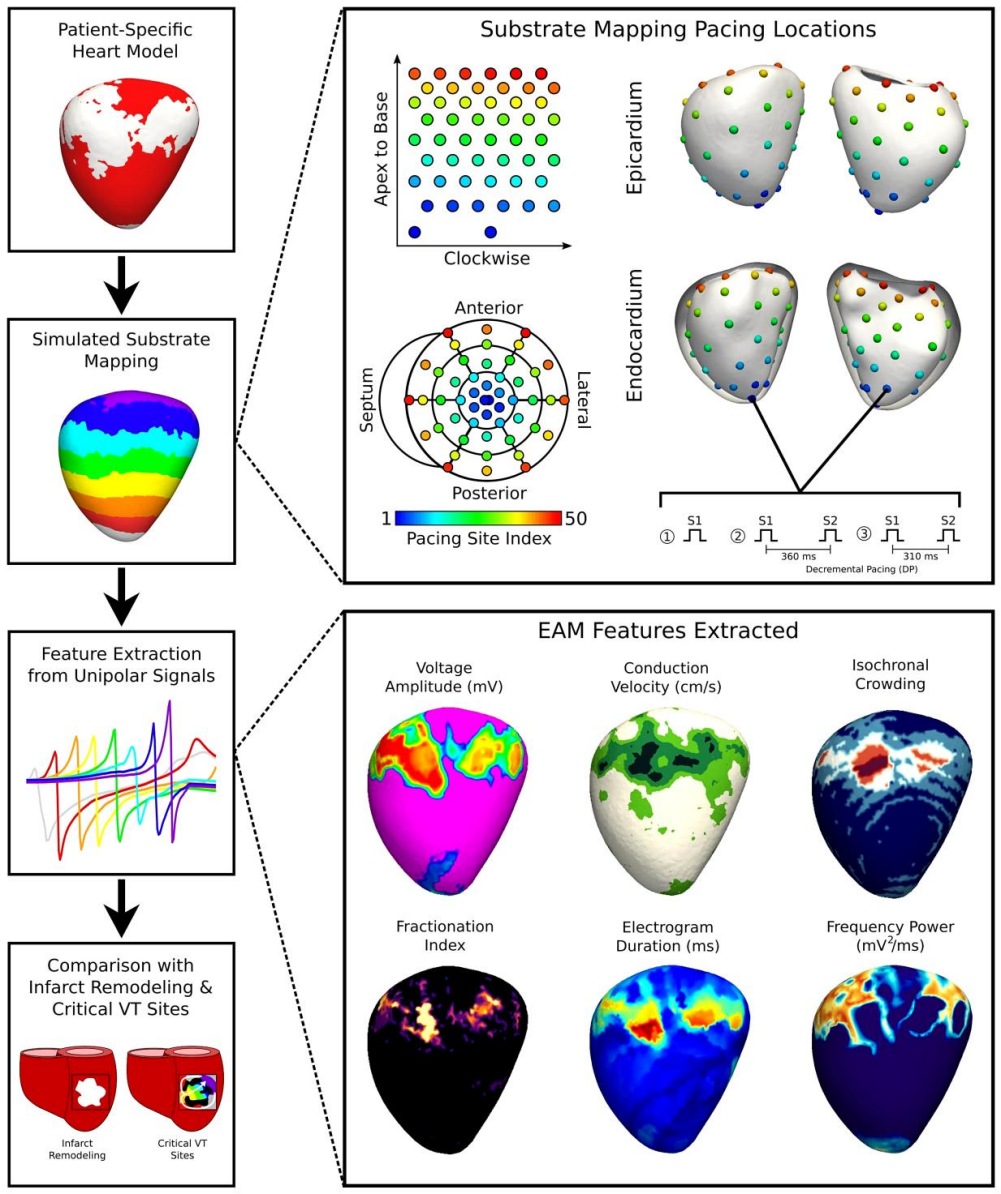
Study workflow. In personalized 3D computational heart models, pacing sites were selected throughout the left ventricle on both the endocardium and epicardium. Three pacing protocols were delivered: (i) an initial S1, (ii) S2 delivered 360 ms after S1, and (iii) S2 delivered 310 ms after S1. Unipolar signal electrograms were simulated along the myocardial surface and substrate EAMs were constructed. From the unipolar signals, six EAM features were extracted: voltage amplitude, conduction velocity, isochronal crowding, fractionation index, electrogram duration, and frequency power. The distributions of these EAM features were then compared with the infarct remodelling distribution and the locations of critical VT sites. EAM, electroanatomic mapping; VT, ventricular tachycardia.

*Figure 1* summarizes the workflow for our computational study. Using patient-specific, computational heart models (*Figure 1*, left, first panel), we simulated unipolar electrogram signals along the myocardial surfaces and constructed substrate EAMs (*Figure 1*, left, second, and third panels). For each heart model, 100 EAMs were constructed, each corresponding to one of 100 pacing locations evenly distributed throughout the endocardial and epicardial surfaces (*Figure 1*, top right). For each pacing experiment, six EAM features were automatically extracted (*Figure 1*, bottom right). Then, we quantified whether these EAM features predicted the local presence of infarct remodelling and critical VT sites (*Figure 1*, left, fourth panel).

### Reconstruction of heart models

Forty-eight personalized, computational 3D ventricular models were reconstructed using imaging scans obtained from post-infarct patients who underwent VT ablation (details in Supplemental material online, *Materials*). Only the left ventricle (LV) was reconstructed for each patient because infarcts predominantly affect the LV, not the right ventricle (RV). To execute simulations of ventricular electrical activity, finite-element, volumetric meshes were reconstructed as done in our previous studies.^11,12^ Fibre orientations, specific to each individual heart model, were assigned to each mesh using a validated rule-based method as in previous publications.^11,12^

From imaging, patient-specific, infarct remodelling distributions, defined as both dense infarcted tissues and border zones, were mapped onto each ventricular model using previously described methodologies (details about myocardial substrate identification on imaging in Supplementary material online, *Materials*).^11,12^ The infarct distribution across all heart models is presented in Supplementary material online, *Figure S1*. Electrophysiological properties for both non-injured myocardium and infarct-remodelled tissues (dense infarct and border zones) were assigned the same parameters as in our previous publications.^11,12^

### Assessment of heart model ventricular tachycardia circuits

Ventricular tachycardias were induced in all ventricular models, using a validated protocol, to determine the locations of critical VT sites in the substrate.^11,12^ Rapid pacing was applied from 17 automatically selected locations in each heart model to identify all possible VTs.^11,12^ Ventricular tachycardias were defined to be re-entrant activity of at least two cycles at the same location, the same definition as in previous publications.^11^

For all VTs in the heart models, exit site locations were identified manually by visually assessing the 3D activation sequence. From the VT exit site, the VT circuit was defined by using the universal ventricular coordinate (UVC) system.^13^ The UVC system maps each heart geometry to a common reference space invariant of its size using three axes: apicobasal (apex to base), transmural (endocardium to epicardium), and rotational (counter-clockwise starting from the lateral myocardium). Using this UVC system, we defined the VT circuit to be the window around the VT exit site; specifically, we used a window of 0.4 units for the apicobasal axis, 72° for the rotational axis, and the entire transmural axis to encompass the whole activation sequence. The VT activation sequence was divided into eight discrete, equally spaced windows (isochrones), beginning with the activation time at the exit site and ending with the activation from the next re-entrant cycle at the exit site. This definition was designed to match a previous clinical definition used for VT circuits.^14^

### Determining pacing locations for substrate mapping

To simulate substrate EAM, pacing was performed from various locations throughout the heart. Pacing locations across heart geometries were standardized by using the UVC system.^13^ Fifty evenly spaced locations on the endocardium and epicardium were selected for a total of 100 pacing sites (*Figure 1*, top right). Although we did not explicitly represent the RV geometry in our heart models, the locations for RV pacing, which is commonly performed in clinical practice, were approximated via the epicardial pacing locations along the LV septum. From each location, an initial current stimulus (S1) was delivered to simulate a paced rhythm in the ventricle. From the same pacing locations, two sets of experiments with an extra-stimulus (S2) were delivered to assess the effects of DP. The extra-stimulus was delivered either at 360 (S2_360ms_) or 310 ms (S2_310ms_), respectively, after the initial S1 (*Figure 1*, top right). With 48 heart models, 100 pacing sites per heart model, and three different stimulus protocols per site, 14,400 pacing simulations were conducted in parallel.

### Simulated substrate electroanatomic mapping during paced rhythms

For each pacing experiment, we simulated high-resolution substrate EAM. First, EAM surface geometries, spanning both epicardium and endocardium, were created using custom software. Triangular surface meshes were created with an edge length resolution of 2.012 ± 0.003 mm. Across EAM surface geometries, there was an average of 13959 ± 2429 points. At each point, unipolar signals were computed using Poisson’s equation, which couples the cellular-level transmembrane potentials in the myocardial volume to extracellular unipolar signals.^15^ These signals were sampled at a frequency of 400 Hz. *S1 maps* and *S2 maps* were defined as the EAMs resulting from S1 and S2 pacing, respectively.

### Feature extraction from unipolar signals during paced rhythms

For all simulated EAMs, six characteristics were computed from the unipolar signals along the surface geometry for both S1 and S2 maps (*Figure 1*, bottom right). For this study, these six characteristics will be referred to as *EAM features*. Those computed from S1 pacing will be referred to as *S1 EAM features* and those from S2 pacing as *S2 EAM features*.

1. Voltage amplitude (*V*_amp_) in mV: This is the most common measurement obtained during EAM procedures.^1^ It is the difference between the maximum and minimum voltage deflection.
2. Conduction velocity (CV) in cm/s: CV was defined per element using the triangulation technique, as described previously.^16^ The CV at each mesh point was defined as the average of CVs across elements within a radius of 5 mm.
3. Isochronal crowding (IC): This metric is used in contemporary ablation procedures and indicates activation slowing.^17^ Sixteen equally spaced time windows (isochrones) were defined between the earliest and latest activation times for each pacing. For each point on the EAM geometry, the number of isochrones present within a 1 cm radius was computed.
4. Fractionation index (FI): The FI is a measure of electrogram fractionation, computed by an algorithm.^15^ Fractionation index estimates the number of deflections within the unipolar signal by fitting a smoothed ‘normal’ signal template. A lower FI indicates normal electrical wave propagation, whereas a higher FI indicates more irregular conduction and could be indicative of critical VT sites.
5. Electrogram duration (EGM_dur_) in ms: EGM_dur_ was computed using a previously described method.^18^ A sliding window of 27.5 ms was defined around each time point and moved across the whole signal. Within each window, the standard deviation (SD) was computed. The EGM_dur_ was defined as the difference between last and first-time windows whose SDs exceeded 10% of the maximum SD across all windows. Longer EGM_dur_ has previously been shown to correlate with critical VT sites.^19^
6. Frequency power (FP), in mV^2^/ms: FP was defined as the area under the power spectrum of the unipolar signal. This measurement has previously been shown to be predictive of intramural inexcitable substrate.^20^

### Concordance between electroanatomic mapping features on different paced electroanatomic mapping maps

The concordance of S1 EAM features between different maps was measured. We define the term *EAM feature concordance* as the correlation between EAM features from two different maps. A higher EAM feature concordance means that two paced maps were similar whereas a lower value means that two maps were dissimilar. Electroanatomic mapping feature concordances were compared using an unpaired *t*-test.

### Defining the infarct remodelling on the electroanatomic mapping surface geometry

To compare 2D surface EAM features with 3D infarct remodelling from imaging, we first projected each heart’s infarct distribution onto the surface geometry. Each point on the EAM surface was labelled as being in (i) non-injured myocardium or (ii) infarct remodelling. An EAM point was defined as being within the infarct remodelling if at least 10% of the surrounding myocardium within a 5 mm radius consisted of infarcted tissue. To assess whether this threshold definition would impact our results, we performed an additional sensitivity analysis by varying our threshold definition for infarct remodelling from a 10% cutoff to a 50% cutoff, the results of which are presented in Supplementary material online, *Figure S2*. Ultimately, the choice of threshold definition did not significantly impact the findings of this study (see Supplementary material online, *Figure S2*). All points not in infarct remodelling were considered to be in non-injured myocardium. We divided our cohort of 48 personalized heart models into two groups for further analysis based on the median remodelling percentage across the cohort: (i) hearts with larger amounts of infarct remodelling (hLIR) above the median and (ii) hearts with smaller amounts of infarct remodelling (hSIR) below the median. For our cohort, the median percentage of infarct remodelling across hearts was 28.5% (IQR: 22.3% 35.5%).

For each heart, we computed the geodesic distances along the EAM surface geometry between a given pacing location and each point in the zone of infarct remodelling. These distances were averaged to derive a single estimate of how far the given pacing location was from the infarct. This process was repeated for every pacing location in the heart. Using these values, we uniquely ranked the pacing locations in each heart in order of most proximal (1) to most distal (100) from the infarct.

### Using electroanatomic mapping features to predict the presence of infarct remodelling

For each heart and each of the six EAM features, we evaluated how well S1 EAM features from a single map predicted the presence of infarct remodelling from imaging at each point on the EAM surface geometry. To do so, we used a simple logistic regression model. We then evaluated whether, for each heart, incorporating S1 EAM features from multiple maps paced at different locations would be more strongly predictive of whether a given point on the EAM surface geometry was in infarct remodelling. To do this, we built 100 multivariable logistic regression models for each of the six EAM features. For each regression model, S1 EAM features from different maps were added as covariates. Electroanatomic mapping features were added sequentially from maps starting first with those paced most proximally to the infarct (1) and ending with those paced most distally (100). This means that for a given heart model, the *N*^th^ multivariable regression model had *N* covariates: the EAM features from the *N* most proximally paced S1 maps.

Next, we assessed whether adding S2 EAM features from maps generated by DP at a single location would improve detection of infarct remodelling at a point on the EAM surface geometry. For each pacing location in each heart model, three sets of multivariable logistic regression models were built to quantify how well S2 EAM features predicted whether a point was within infarct remodelling using different covariates: EAM features from (i) S1 and S2_360ms_ maps, (ii) S1 and S2_310ms_ maps, and (iii) S1 and both S2 maps.

Lastly, we investigated whether adding S2 EAM features from multiple maps paced at different locations would further improve detection of infarct remodelling at a point. For each heart, multivariable logistic regression models were constructed with the covariates being EAM features from either (i) multiple S1 and S2_360ms_ maps, (ii) multiple S1 and S2_310ms_ maps, or (iii) multiple S1, S2_360ms_, and S2_310ms_ maps. For each regression model, EAM features were sequentially incorporated as covariates, beginning with EAM features from maps generated by pacing sites most proximal to the infarct and ending with maps generated by pacing sites most distal to the infarct.

### Projection of ventricular tachycardia activation sequence onto electroanatomic mapping surface geometry

Next, we compared, in areas where VTs perpetuate, EAM features and the spatial structure of the VT circuit. Because the VT circuit has a 3D activation sequence involving the intramural myocardium, its structure could not be directly compared with the EAM features on the 2D surface geometry. Thus, we first projected the 3D VT circuit activation sequence onto the EAM geometry surface. For each EAM surface point within the area of the VT circuit, its activation was defined as the most frequently occurring isochrone in the myocardium within a 5 mm radius. The distance between each substrate mapping pacing location and each VT circuit’s exit site was also computed to approximate how far each given pacing location was from the VT circuit. All 100 pacing locations were then ranked in order of most proximal (1) to most distal (100) from the VT circuit.

We determined whether EAM features in areas where VTs manifest could distinguish between the outer and inner parts of the VT circuit. As we define our VT circuit to begin at the VT exit site, the initial half of the activation sequence represents propagation through the outer part of the VT circuit, whereas the latter half represents propagation through the inner part of the circuit.^14^ Thus, the *outer circuit* was defined as points on the EAM surface geometry that were activated during the first four isochrones. The *inner circuit* was defined as points along the EAM surface that were activated during the last four isochrones. The inner circuit contains the *critical VT site* that is searched for intraprocedurally and represents the ideal target for ablation.^21^ The outer and inner parts of each VT circuit were identified on both the endocardium and epicardium. From this, we delineated four subregions per VT: endocardial outer, endocardial inner, epicardial outer, and epicardial inner VT circuit components.

### Using electroanatomic mapping features to predict the locations of critical ventricular tachycardia circuit components

We evaluated whether EAM features in each VT subregion could distinguish whether that subregion was part of the critical inner VT circuit or the outer circuit. For each VT, EAM features recorded in the four VT subregions (the endocardial outer, endocardial inner, epicardial outer, and epicardial inner) were averaged. Logistic regression models were then built with the covariates being EAM features averaged within one of the four VT subregions, and the dependent variable being 0 (if outer circuit) or 1 (if inner circuit).

Two sets of multivariable logistic regression models were developed using different covariates to determine if pacing closer to a VT circuit would improve identification of the critical VT site on EAM. The first and second sets used data from maps generated by (i) the 50 pacing locations most proximal to the VT circuit and from (ii) the 50 pacing locations most distal to the VT circuit, respectively. To predict whether a given VT subregion was part of the outer or inner VT circuit, for both sets of models, the *N*^th^ regression model incorporated, as covariates, S1 and S2 EAM features from maps generated by the *N* pacing sites located either (i) most proximally to the VT for the first set or (ii) most distally from the VT for the second set.

### Evaluation metrics for regression models

For all regression models, four evaluation metrics were computed: (i) area under the receiver operating characteristic (AUROC) which varies from 0.5 to 1, (ii) F1 score (the harmonic mean between precision and recall that varies from 0 to 1), (iii) sensitivity, and (iv) specificity. For all metrics except for AUROC, a default threshold probability of 0.5 was used as the cutoff for the regression model. When detecting infarct remodelling, we focused on the F1 score because of the class imbalance between the number of points labelled as non-injured myocardium vs. infarct remodelling. F1 scores between different sets of heart models were compared using a paired *t*-test. For identifying the critical VT site, we focused on the AUROC since there were a balanced number of inner and outer VT circuit components.

## Results

We first evaluated which pacing locations in each personalized heart model successfully captured because the pacing sites could have been placed in non-conducting tissue. 4484/4800 (93.4%) of all S1 pacing stimuli, 4422/4800 (92.1%) of all S2_360ms_ pacing stimuli, and 4259/4800 (88.7%) of all S2_310ms_ pacing stimuli successfully resulted in electrical propagation. This discrepancy between the number of successful S1 and S2 pacing simulations resulted from tissue at the pacing site still being refractory by the time the extra-stimulus was delivered.

### Concordance of S1 electroanatomic mapping features from different pacing locations

**Figure 2:**
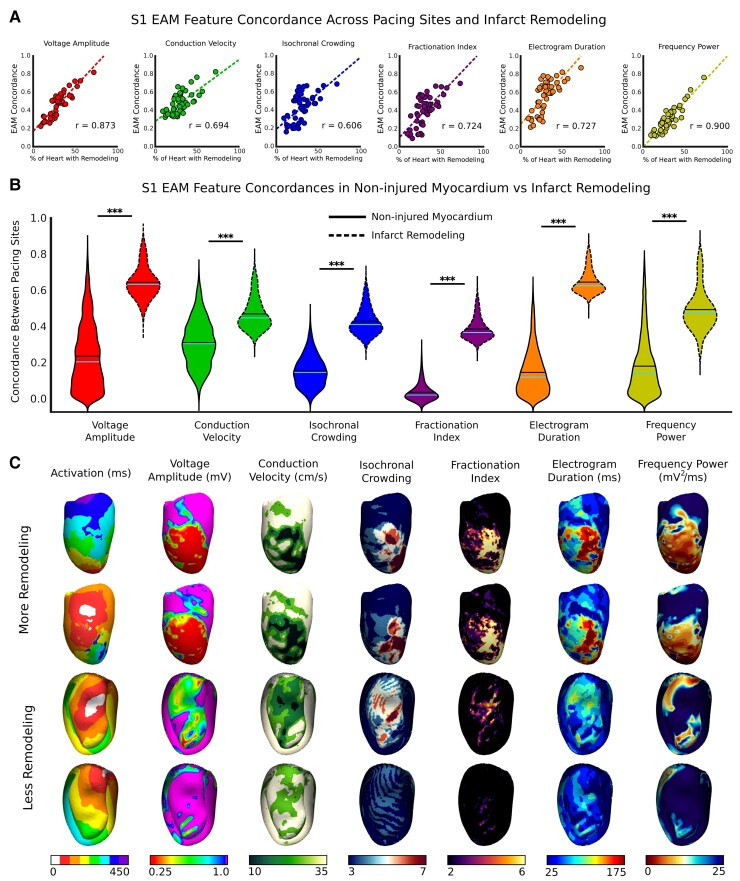
Concordance between S1 EAM features from different paced maps. (*A*) Relationship between percentage remodelling and EAM feature concordance. Colours represent the different EAM features; dashed line is the line of best fit. (*B*) Violin plots illustrating the EAM feature concordances in non-injured myocardium vs. infarct remodelling. (*C*) Example depicting EAM feature concordance in a heart with more remodelling vs. a heart with less remodelling. EAM, electroanatomic mapping.

We first characterized how the amount of infarct remodelling affects concordance between S1 EAM features on maps paced at different locations. *Figure 2A* shows the relationship between the amount of infarct remodelling and S1 EAM feature concordance. There was overall a strong correlation between the infarct remodelling as a percentage of volume and S1 EAM feature concordances (*V*_amp_: *r* = 0.873, CV: *r* = 0.694, IC: *r* = 0.606, FI: *r* = 0.724, EGM_dur_: *r* = 0.727, FP: *r* = 0.900; *P* < 0.0005 for all) (*Figure 2A*). This indicates that different activation wavefronts are more likely to change EAM substrate characterization in hearts with less remodelling.

The S1 EAM feature concordances across different maps for points in infarct remodelling vs. points in non-injured myocardium are shown in *Figure 2B*. Electroanatomic mapping feature concordance was higher between different maps when the distance between their pacing sites was smaller (*V*_amp_: *r* = −0.39, CV: *r* = −0.80, IC: *r* = −0.77, FI: *r* = −0.81, EGM_dur_: *r* = −0.53, FP: *r* = −0.49). S1 EAM feature concordance was significantly higher for points in infarcted remodelling than in non-injured myocardium (*V*_amp_: 0.65 vs. 0.17, CV: 0.48 vs. 0.32, IC: 0.44 vs. 0.16, FI: 0.40 vs. 0.04, EGM_dur_: 0.66 vs. 0.11, FP: 0.50 vs. 0.15; unpaired *t*-test, *P* < 0.0005 for all) (*Figure 2B*). This means that EAM characterization of the infarct distribution is less sensitive to the effects of activation wavefront directionality.

*Figure 2C* illustrates S1 EAM features from two different maps in two hearts: one hLIR (first two rows) and one hSIR (last two rows). Electroanatomic mapping features were more concordant for hLIR than for hSIR, particularly within the infarct remodelling (*Figure 2C*). Thus, the use of MWP in substrate EAM characterization would likely be more advantageous in hearts with smaller amounts of remodelling than in hearts with larger amounts of remodelling.

### Detection of infarct remodelling using electroanatomic mapping features from a single-paced S1 map

**Figure 3:**
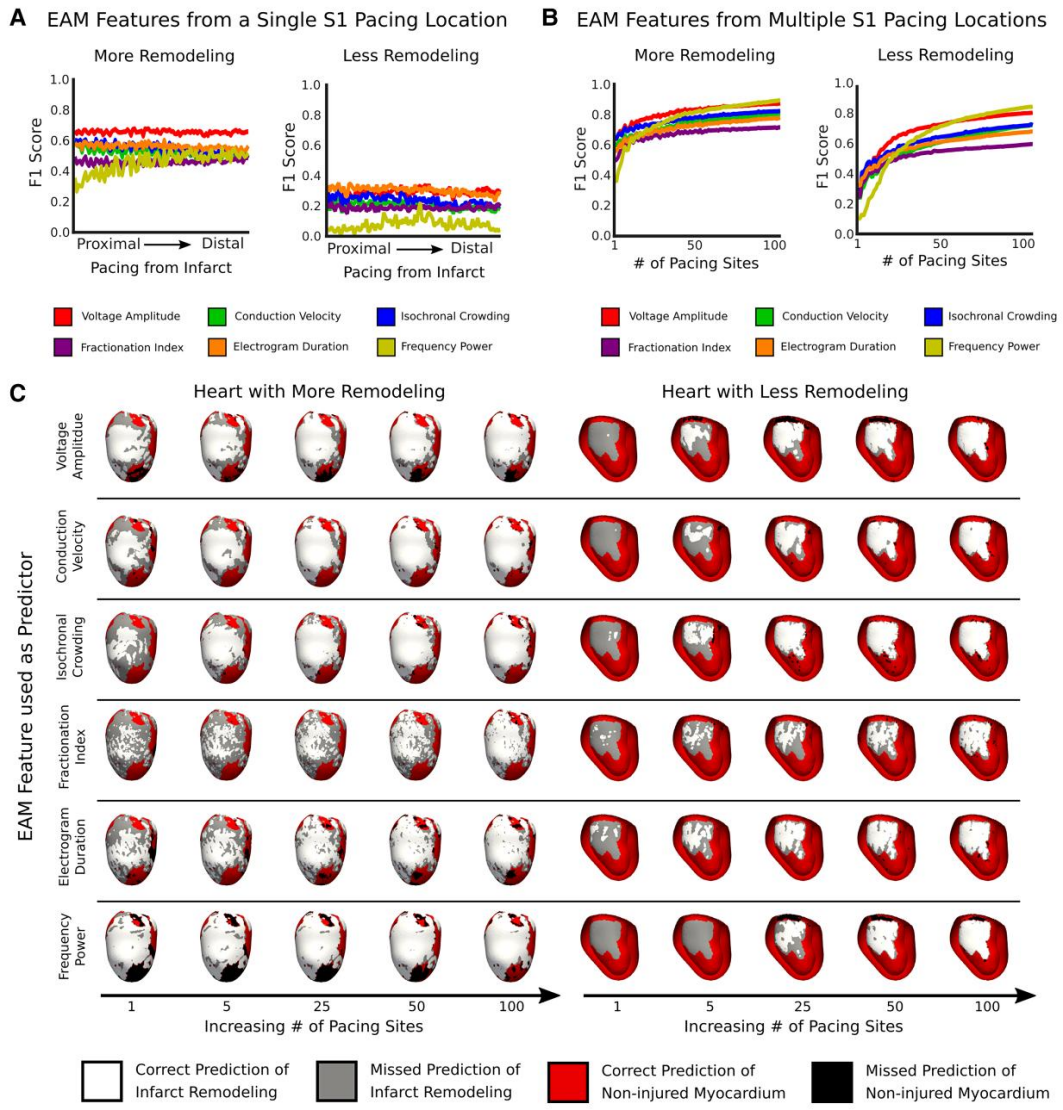
Detection of infarct remodelling using S1 EAM features. F1 scores for logistic regression models predicting the presence of infarct remodelling using EAM features from (*A*) individual and (*B*) multiple pacing locations in hearts with more (left) vs. less remodelling (right). (*C*) Example of how well S1 EAM features from multiple maps identifies the infarct distribution in a heart with more vs. less remodelling. EAM, electroanatomic mapping.

We next assessed whether S1 EAM features from a map generated by pacing from a single site could be used to accurately detect the local presence of infarct remodelling. *Figure 3A* summarizes the results from regression models which utilize S1 EAM features from different maps. The ability of local S1 EAM features to detect presence of infarct remodelling did not significantly depend on distance of pacing location from the infarct distribution (*Figure 3A*). S1 EAM features in hLIR achieved higher F1 scores than those in hSIR (*V*_amp_: 0.64 vs. 0.28, CV: 0.51 vs. 0.19, IC: 0.53 vs. 0.23, FI: 0.45 vs. 0.18, EGM_dur_: 0.55 vs. 0.30, FP: 0.44 vs. 0.07; paired *t*-test, *P* < 0.0005 for all). Thus, in hearts with smaller amounts of remodelling, EAM features from S1 maps generated by a single pacing site poorly detect infarct remodelling, regardless of pacing location.

### Detection of infarct remodelling using electroanatomic mapping features from multiple paced S1 maps

Given that S1 EAM features from maps generated by a single pacing site poorly predicted infarct remodelling, we next assessed whether S1 EAM features from maps generated by MWP would improve detection of infarct remodelling. Performance metrics of regression models which use S1 EAM features from multiple maps to identify infarct remodelling in hLIR and hSIR are shown in *Figure 3B*. Incorporating S1 EAM features from maps generated by MWP improved detection of infarcted remodelling for both hLIR and hSIR. F1 scores substantially improved when incorporating S1 EAM features from 1 vs. 25 maps for hLIR (*V*_amp_: 0.64 vs. 0.79, CV: 0.60 vs. 0.70, IC: 0.64 vs. 0.74, FI: 0.50 vs. 0.66, EGM_dur_: 0.56 vs. 0.69, FP: 0.38 vs. 0.73; *P* < 0.0005 for all) (*Figure 3B*, left panel). In hSIR, even greater improvements in F1 scores occurred when using S1 EAM features from 1 vs. 25 maps (*V*_amp_: 0.31 vs. 0.66, CV: 0.26 vs. 0.54, IC: 0.32 vs. 0.59, FI: 0.24 vs. 0.51, EGM_dur_: 0.33 vs. 0.56, FP: 0.10 vs. 0.56; *P* < 0.0005 for all) (*Figure 3B*, right panel). This means that MWP improves detection of infarct remodelling on EAM and that this improvement is considerably larger in hearts with less remodelling.

With EAM features from 100 paced S1 maps, F1 scores in both hLIR and hSIR both plateaued (*V*_amp_: 0.87 vs. 0.81, CV: 0.81 vs. 0.73, IC: 0.82 vs. 0.73, FI: 0.72 vs. 0.60, EGM_dur_: 0.78 vs. 0.69, FP: 0.90 vs. 0.86) (*Figure 3B*). Thus, although MWP improves detection of underlying remodelling, there are diminishing gains in generating EAMs from too many pacing locations.

*Figure 3C* provides two examples of using the different EAM features to detect infarct remodelling in heart models. For the heart with greater amounts of remodelling, EAM features even from a single pacing location successfully identified most of the infarct distribution (*Figure 3C*, left). However, for the heart with smaller amounts of remodelling, more pacing sites were needed to accurately identify the infarct distribution (*Figure 3C*, right). As the number of pacing locations increased from 25 to 100, there was little improvement in the detection of infarct remodelling for both the hLIR and hSIR (*Figure 3C*).

### Impact of decremental pacing from individual sites on detection of remodelling

**Figure 4:**
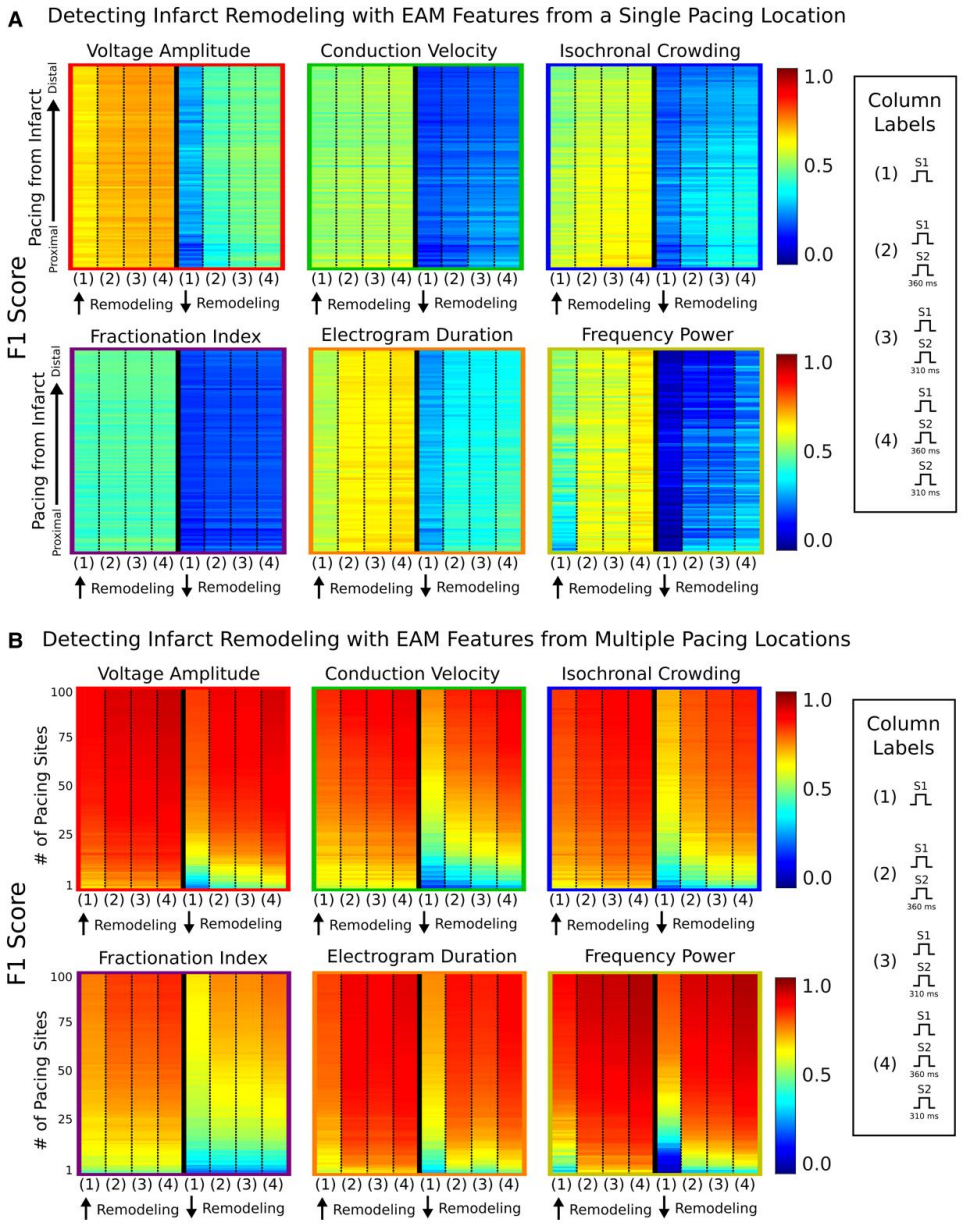
Impact of decremental pacing on detection of infarct remodelling. F1 scores for logistic regression models predicting the presence of infarct remodelling using EAM features from both S1 and S2 maps paced from (*A*) individual and (*B*) multiple locations. Electroanatomic mapping features were ordered based on distance from infarct, starting with maps paced most proximally and ending with maps paced most distally (bottom to top). EAM, electroanatomic mapping.

We also investigated if maps created from DP at a single location would improve detection of infarct remodelling. *Figure 4A* shows the evaluation metrics of using EAM features from (i) only S1, (ii) S1 and S2_360ms_, (iii) S1 and S2_310ms_, and (iv) S1 and both S2 maps in hLIR and hSIR. In hLIR, adding S2 EAM features modestly improved the F1 score [(S1 only vs. adding S2_360ms_ vs. adding S2_310ms_): *V*_amp_: 0.63 vs. 0.68 vs. 0.69, CV: 0.50 vs. 0.51 vs. 0.52, IC: 0.52 vs. 0.57 vs. 0.58, FI: 0.44 vs. 0.45 vs. 0.45, EGM_dur_: 0.54 vs. 0.62 vs. 0.64, FP: 0.44 vs. 0.57 vs. 0.57; *P* < 0.0005 for all] (*Figure 4A*). In hSIR, there were also improvements in F1 scores when adding S2 EAM features [(S1 only vs. adding S2_360ms_ vs. adding S2_310ms_): *V*_amp_: 0.28 vs. 0.44 vs. 0.47, CV: 0.19 vs. 0.21 vs. 0.23, IC: 0.22 vs. 0.29 vs. 0.33, FI: 0.18 vs. 0.20 vs. 0.20, EGM_dur_: 0.29 vs. 0.38 vs. 0.38, FP: 0.07 vs. 0.20 vs. 0.20; *P* < 0.0005 for all]. In all hearts, using EAM features from both S2 maps resulted in similar F1 scores comparable to that of S2_360ms_ or S2_310ms_ maps alone (*Figure 4A*). This indicates that DP from a single location only modestly improves detection of underlying infarct remodelling, and mapping with multiple extra-stimuli using different coupling intervals has little added benefit for substrate characterization on EAM.

### Effects of decremental pacing from multiple sites on detection of remodelling

We next assessed if combining DP and MWP would synergistically improve detection of infarct remodelling and thus, require a smaller number of EAM maps to achieve the same level of detection accuracy. We summarize the results of infarct remodelling detection using S1 and S2 EAM features from multiple maps in *Figure 4B*. With the combination of DP and MWP, fewer maps were required to detect infarct remodelling at a similar performance as compared with that of using MWP only. For instance, in hLIR, EAM features from five paced S1 maps achieved F1 scores of 0.67 for *V*_amp_, 0.59 for CV, 0.63 for IC, 0.55 for FI, 0.60 for EGM_dur_, and 0.45 for FP. However, EAM features derived from only one S1 and one S2_310ms_ map (for a total of two maps) achieved similar (*V*_amp_: 0.69, EGM_dur_: 0.63, FP: 0.66; *P* > 0.05) or better F1 scores (CV: 0.59, IC: 0.64, FI: 0.50; *P* < 0.0005) except for FI (0.50). Similarly, in hSIR, most EAM features from one S1 and one S2_310ms_ map achieved similar F1 scores than those from 5 paced S1 maps (*V*_amp_: 0.41 vs. 0.45, CV: 0.22 vs. 0.20, IC: 0.34 vs. 0.26, EGM_dur_: 0.41 vs. 0.38, FP: 0.31 vs. 0.06; *P* < 0.0005 for FP, *P* > 0.05 for all other EAM features) except for FI (0.21 vs. 0.29). Thus, no matter the amount of infarct remodelling in the heart, combining MWP and DP improves EAM substrate characterization to a greater extent than that of MWP or DP alone.

### Relationship between electroanatomic mapping features and ventricular tachycardia circuits

Our next goal was to assess the relationship between VT circuits and EAM features. One hundred and ninety-two VTs were induced across the 48 heart models (4.0 ± 3.1 VTs). The inner VT circuit component was present on both the epicardium and endocardium in 167/192 VTs, only the epicardium in 20/192 VTs, only the endocardium in 4/192 VTs, and purely mid-myocardial in 1/192 VTs.

**Figure 5:**
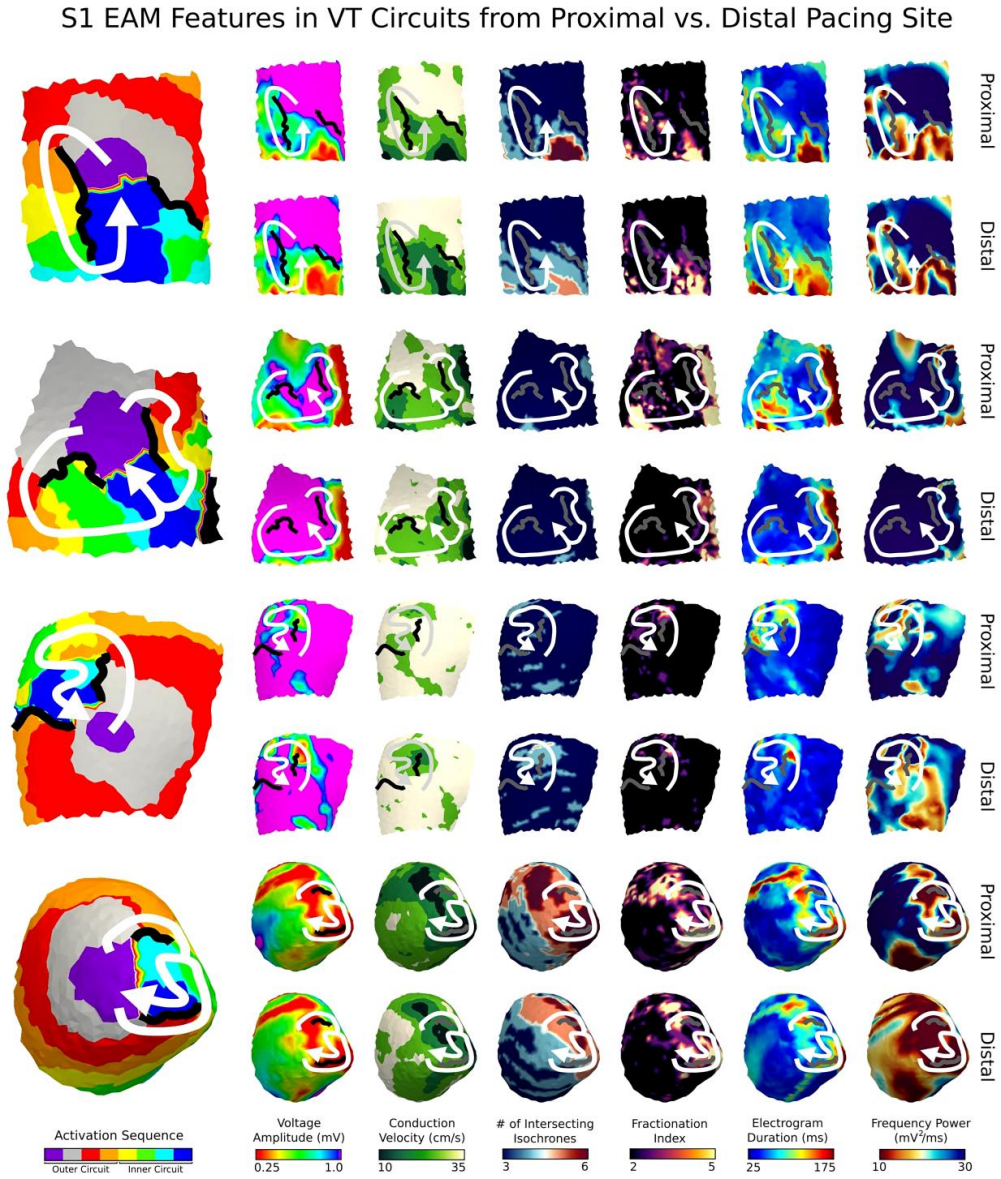
Examples of VT circuits along with S1 EAM features. For each example shown, the top and bottom rows display the S1 EAM features from a proximally and distally paced map, respectively. White/light grey arrows indicate the re-entrant pathway of the VT circuit, connecting from the exit site to the end of the common pathway. The black/dark grey lines indicate the lines of functional conduction block. VT, ventricular tachycardia; EAM, electroanatomic mapping.

The inner VT circuit exhibited lower voltage, greater conduction slowing, greater fractionation, prolonged electrogram duration, and lower FP during S1 paced rhythms than that of the outer VT circuit (*V*_amp_: 0.84 ± 0.08 vs. 0.96 ± 0.10 mV, CV: 26.3 ± 1.7 vs. 28.9 ± 2.3 cm/s, IC: 3.6 ± 0.2 vs. 3.3 ± 0.2, FI: 2.6 ± 0.1 vs. 2.3 ± 0.1, EGM_dur_: 74.0 ± 4.4 vs. 63.3 ± 4.1 ms, FP: 24.7 ± 2.7 vs. 29.2 ± 3.5 mV^2^/ms). S1 EAM features in VT circuits were heterogeneous across different hearts and across different maps within the same heart. *Figure 5* shows examples of VT circuits along with the corresponding S1 EAM features from maps generated by pacing from two sites: one proximal and one distal from the VT circuit. In these examples, S1 EAM features from maps paced proximally to VT circuits accentuated differences between the outer and inner VT circuit better than EAM features from maps paced distally (*Figure 5*). Thus, EAM features from maps paced closer to a VT circuit may be useful for distinguishing the outer vs. inner VT circuit.

### Identifying the inner ventricular tachycardia circuit using S1 electroanatomic mapping features

**Figure 6:**
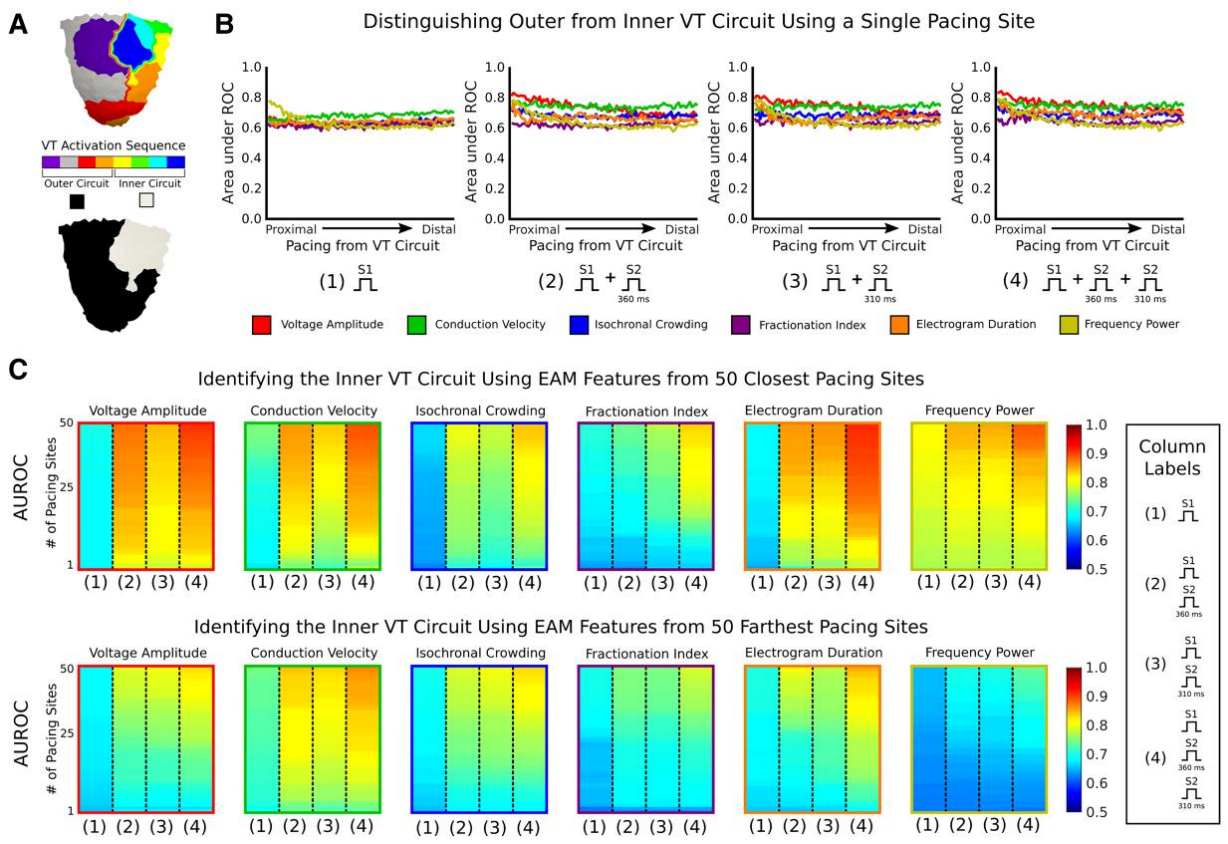
Identifying the inner VT circuit using EAM features. (*A*) Example of how outer (black) and inner (white) VT circuits are defined. Area under the receiver operating characteristics for logistic regression models identifying the inner VT circuit using EAM features from (*B*) a single pacing site and (*C*) multiple pacing sites. Top and bottom for (*C*) show the results from when EAM features from the 50 maps paced most proximally and 50 maps paced most distally to the VT circuit were used, respectively. VT, ventricular tachycardia; EAM, electroanatomic mapping.

We hypothesized that the EAM pacing location relative to the zone where the VT perpetuates might have an impact on whether the resultant EAM features could correctly identify critical VT sites. Thus, we next investigated how well EAM features of maps paced from different locations distinguished the outer from the inner VT circuit, the critical site for ablation targeting. *Figure 6A* illustrates the definition of the outer vs. inner VT circuitry. In *Figure 6B*, we summarize how well EAM features of maps paced from a single location identified the inner VT circuit. S1 EAM features from a single map poorly discriminated the outer from inner VT circuit [(AUROC), *V*_amp_: 0.63, CV: 0.68, IC: 0.63, FI: 0.61, EGM_dur_: 0.64, FP: 0.63]. Contrary to our hypothesis, distance of pacing site from the VT circuit did not significantly impact the predictive capabilities of S1 EAM features in identifying the critical VT site (*Figure 6B*).

With S1 EAM features from multiple maps, identification of the critical inner VT circuit only marginally improved. *Figure 6C* presents the results for when EAM features from multiple maps were added; the two sides show the metrics in cases when maps from the 50 most proximal pacing sites (top) vs. those from the 50 most distal pacing sites (bottom) were added. When S1 EAM features for 1 vs. 50 proximal pacing sites were compared, the AUROC only improved modestly (*V*_amp_: 0.67 vs. 0.70, CV: 0.67 vs. 0.75, IC: 0.62 vs. 0.67, FI: 0.63 vs. 0.71, EGM_dur_: 0.64 vs. 0.69, FP: 0.77 vs. 0.81) (*Figure 6C*, top, first column of each panel). Similarly, incorporation of EAM features from the single most distal vs. 50 distally paced maps also only marginally improved the AUROCs (*V*_amp_: 0.66 vs. 0.68, CV: 0.70 vs. 0.75, IC: 0.65 vs. 0.70, FI: 0.62 vs. 0.70, EGM_dur_: 0.66 vs. 0.70, FP: 0.63 vs. 0.65) (*Figure 6C*, bottom, first column of each panel). Hence, substrate mapping with different activation wavefront directionalities alone does not significantly improve identification of the inner VT circuit.

### Improving inner ventricular tachycardia circuit identification with decremental pacing

Although S1 EAM features poorly predicted the critical VT site, we hypothesized that inclusion of S2 EAM features might improve identification of the inner VT circuit. First, we evaluated whether DP from different pacing locations would improve detection of critical VT sites. Inclusion of S2 EAM features on a map generated from a single pacing site improved identification of the inner VT circuit [(AUROC) S1 vs. adding S2_360ms_ vs. adding S2_310ms_, *V*_amp_: 0.63 vs. 0.73 vs. 0.74, CV: 0.68 vs. 0.74 vs. 0.74, IC: 0.63 vs. 0.68 vs. 0.68, FI: 0.61 vs. 0.62 vs. 0.63, EGM_dur_: 0.64 vs. 0.66 vs. 0.67, FP: 0.63 vs. 0.64 vs. 0.64] (*Figure 6B*). Unlike with S1 EAM features alone, the improved predictive capacity of these additional S2 EAM features was dependent on the distance between the VT circuit and the pacing location (*Figure 6B*). The majority of S2 EAM features from the maps paced most proximally to the VT circuit achieved higher AUROCs than those from the maps paced most distally [(S2_360ms_) *V*_amp_: 0.81 vs. 0.69, EGM_dur_: 0.71 vs. 0.65, FP: 0.77 vs. 0.62; (S2_310ms_) *V*_amp_: 0.79 vs. 0.69, EGM_dur_: 0.77 vs. 0.67, FP: 0.79 vs. 0.62], whereas the remaining EAM features had moderate to minimal differences in AUROC [(S2_360ms_) CV: 0.76 vs. 0.75, IC: 0.72 vs. 0.74, FI: 0.63 vs. 0.63; (S2_310ms_) CV: 0.72 vs. 0.74, IC: 0.69 vs. 0.69, FI: 0.66 vs. 0.63] (*Figure 6B*). Hence, this indicates that during EAM, DP should be delivered at locations proximal to the suspected VT circuit to improve identification of the critical VT sites.

Lastly, we determined whether S2 EAM features from multiple maps would further improve discrimination of outer vs. inner VT circuitry. Electroanatomic mapping with DP decreased the number of paced maps required to attain the same level of accuracy in identifying critical VT sites (*Figure 6C*). When incorporating S2 EAM features from the single most proximally paced location (one S1 and one S2 map), AUROCs were generally higher than those achieved using only S1 EAM features from all 50 proximally paced maps [(S1 vs. S2_360ms_ and S2_310ms_) *V*_amp_: 0.70 vs. 0.81 and 0.79, CV: 0.75 vs. 0.76 and 0.72, IC: 0.67 vs. 0.72 and 0.69, EGM_dur_: 0.69 vs. 0.71 and 0.77] except for FI (0.71 vs. 0.63 and 0.66) and FP (0.81 vs. 0.77 and 0.79) (*Figure 6C*, top). Thus, substrate mapping with a combination of DP and MWP is more effective than just using MWP alone in identifying critical VT sites.

## Discussion

In this study, we systematically assessed, using a personalized computational heart modelling approach, how MWP, DP, and the combination of both impact EAM characterization of the VT substrate. We establish that MWP is more advantageous for substrate EAM in hearts with smaller amounts of infarct remodelling. We demonstrate that EAM with MWP alone more accurately detects infarct remodelling than EAM with DP alone. On the other hand, DP improves identification of critical VT sites more than MWP on EAM, especially when paced close to the suspected VT circuit. Lastly, combined MWP and DP synergistically improves identification of both infarct remodelling and critical VT sites, decreasing the number of maps needed to achieve the same level of detection accuracy. We anticipate that these results will provide guidance on best practices for functional mapping strategies to maximize accuracy of VT substrate characterization.

Optimizing the number and location of pacing sites is vital for efficient functional EAM. Even with high-resolution mapping technological advancements, creating comprehensive substrate maps using multiple paced rhythms are labour-intensive, which constrains the number of maps that can be created intraprocedurally. Studies investigating only MWP^3–5^ have paced from at max three sites: atria (for sinus rhythm), the RV, and the LV. In the various studies investigating only DP,^6–9^ typically two maps (S1 and S2) were acquired. Additionally, these studies chose pacing locations irrespective of the patient-specific remodelling distribution, typically in the right ventricular apex and in the lateral LV.^4^ Unlike these studies, using our personalized computational approach, we could simultaneously examine the individual and combined effects of MWP and DP on EAM. We demonstrated that using MWP and DP in combination instead of just using MWP decreased the number of paced maps necessary for accurate substrate characterization. Furthermore, we demonstrated that the distance of a pacing location relative to a VT circuit modulates the effectiveness of MWP and DP in identifying the critical VT site. Electroanatomic mapping features from multiple maps paced more proximally to a suspected VT circuit were more likely to uncover the critical VT site than EAM features from multiple maps paced distally. Thus, given these results, we recommend that functional EAM strategies be custom-tailored to the patient-specific infarct distribution, prioritizing a combination of MWP and DP at sites proximal to the location of a suspected VT circuit.

Our results also have implications for determining which patients are most likely to benefit from functional EAM strategies. Substrate EAM characterization with MWP is likely to be more beneficial in hearts with smaller amounts of remodelling. Prior evidence suggests that voltage mapping using MWP in hearts with less dense scar patterns showed lower concordance, meaning that identification of remodelling distributions of smaller size is more impacted by activation wavefront directionality.^3^ In our study, all EAM features from maps paced at different locations were consistently more variable in hearts with less remodelling. We also demonstrated that using EAM features from multiple paced maps improved accuracy of detecting infarct remodelling more in hearts with smaller amounts of remodelling than in hearts with a greater burden of remodelling. Collectively, this means that EAM features computed from various wavefront directions offer different and complementary information that when combined can significantly improve detection of infarct remodelling in hearts with smaller burdens of remodelling. These findings are especially important in the context of non-ischemic cardiomyopathy where structural remodelling is often smaller in size, patchy, diffuse, and located in the mid-myocardium/epicardium. For these reasons, the non-ischemic substrate is often difficult to identify on EAM and this constitutes a major reason for post-ablation VT recurrence. Hence, if the extent of remodelling is suspected to be small either based on disease aetiology, pre-procedural imaging, or a lack of low voltage areas on baseline sinus rhythm mapping, then we would recommend that functional EAM be performed with MWP to accurately delineate the substrate and localize critical VT sites.

Our study has several limitations. First, we simulated only unipolar, not bipolar, electrograms. This was intentionally done to isolate the effects of MWP and DP on EAM characterization because various factors are well-known to affect bipolar electrograms such as catheter orientation, electrode size, and interelectrode distances. Second, as done in all our previous predictive modelling studies, electrophysiology incorporated into the models was not patient-specific, due to the need for invasive measurements to acquire that.^10^ Importantly, the use of the same electrophysiological parameters across heart models served to ensure that the effects observed were due to MWP and DP, and not because of different electrical parameters across hearts. However, patient hearts may exhibit electrophysiological heterogeneity across different hearts and even within the same heart (in addition to the heterogeneity induced by the presence of infarcted tissues with altered electrophysiological properties accounted for in the present models), which could affect our results. Future work should investigate the impact of this electrophysiological variability on MWP- and DP-based substrate mapping. Third, although the infarct sizes and distributions were consistent with those described in previous studies, the infarct distributions observed in our patient population may not fully encompass the full range of clinically observed pathologies. Larger, more diverse cohorts may need to be examined in future studies.

In conclusion, we comprehensively assessed how MWP and DP affect substrate characterization on EAM using a cohort of personalized computational heart models. We determined that a combination of MWP and DP should be delivered from locations near suspected VT circuits. We further demonstrated how combining the two pacing strategies synergistically improves detection of structural remodelling and critical VT sites, thus decreasing the number of intraprocedural maps required to achieve an accurate representation of the underlying substrate. Our results highlight the potential of leveraging image-based, personalized computational modelling to provide in-depth, pre-procedural, patient-specific characterization of the VT substrate. We anticipate that this information will help optimize functional EAM strategies and ultimately improve VT ablation efficacy.

## Supplementary material

Supplementary material is available at *Europace* online.

## Supplementary Material

euac140_Supplementary_Data

## Data Availability

The data underlying this article will be shared on reasonable request to the corresponding author.
