# Supplementary material for: Wavefront directionality and decremental stimuli synergistically improve identification of ventricular tachycardia substrate: insights from personalized computational heart models
Source: Europace. 2022 Aug 25;25(1):223–35. doi: 10.1093/europace/euac140 (PMC10103576; doi:10.1093/europace/euac140)
Supplement: euac140_Supplementary_Data [file euac140_supplementary_data.docx]

**Supplemental Information**

**Title: Wavefront Directionality and Decremental Stimuli Synergistically Improve Identification of Ventricular Tachycardia Substrate: Insights from Personalized Computational Heart Models**

**Running Title:** Functional Mapping in Ventricular Tachycardia

**Authors:** Eric Sung BA^1,3^, Adityo Prakosa PhD^1,3^, Stephen Kyranakis^1^, Ronald D. Berger MD/PhD^2,3^, Jonathan Chrispin MD^2,3^, Natalia A. Trayanova PhD^1,3^

1 Department of Biomedical Engineering, Johns Hopkins University, Baltimore, MD, USA

2 Section of Cardiac Electrophysiology, Division of Cardiology, Department of Medicine, Johns Hopkins Hospital, Baltimore, MD, USA

3 Alliance for Cardiovascular Diagnostic and Treatment Innovation, Johns Hopkins University, Baltimore, MD, USA

# Supplemental Methods

**Details about Cardiac Imaging**

24 late gadolinium-enhanced magnetic resonance (LGE-MRI) and 24 contrast-enhanced computed tomography (CE-CT) images were acquired from post-infarct patients who underwent VT ablation at two centers as part of an ongoing prospective clinical study. 18/24 of the LGE-MRI images were 2D with a resolution of 1.3 x 1.3 x 5-8 mm, and the remaining 6/24 LGE-MRI images were 3D with a resolution of 1.3 x 1.3 x 2.5 mm. CE-CT images were acquired at a resolution of 0.428-0.625 x 0.428-0.625 x 1.0-3.0 mm. For all 48 images, the left ventricle was semi-automatically segmented as done in previous works.^1,2^

**Identifying Infarct Remodeling Distributions on Imaging**

Infarct remodeling distributions were identified using previously described methodologies from both LGE-MRI^2^ and CE-CT^1^ and incorporated into each heart model. For LGE-MRI, infarct remodeling included both dense scar and grey zone. Dense scar was identified as any voxel with a signal intensity >50% of the maximum intensity in the ventricular myocardium. Grey zone was defined as voxels with signal intensity of 35-50% of the maximum intensity. For CE-CT, infarct remodeling included both dense infiltrating adipose tissue and fat-myocardium admixture. Dense infiltrating adipose tissue was defined as voxels with Hounsfield units (HU) between -180 and -50 whereas fat-myocardium admixture was defined as voxels with an intensity between -50 and -5 HU. Supplemental Figure 1 shows the infarct remodeling distributions across heart models.

**Electrophysiological Properties in Heart Models**

The patient-specific, infarct remodeling distributions were incorporated into the reconstructed heart models. Regardless of the imaging modality, all heart models consisted of three regions: (1) non-injured myocardium, (2) infarct border zones (grey zone for LGE-MRI models, fat-myocardium admixture for CE-CT models), and (3) dense infarcted tissues (dense scar for LGE-MRI models, dense infiltrating adipose tissue for CE-CT models). Electrophysiological properties for each region were assigned as done in previous works.^1,2^ In non-injured myocardium, conductivity values of 0.08 and 0.009 S/m were assigned to the longitudinal and transverse directions. In grey zone and fat-myocardium admixture, the transverse conductivity was decreased by 90% to represent the electrical changes that occur in infarct border zone regions. Dense scar and dense infiltrating adipose tissue were both modeled as non-conducting tissues.

Ionic properties of non-injured myocardium were represented using the baseline human ventricular myocyte ten Tusscher model. Lastly, in infarct border zones, ionic properties in both regions were adjusted in accordance with experimental literature: the peak I­_Na_ was scaled to 38%, I_CaL_ was scaled to 31%, I­_Kr_ was scaled to 30%, and I_Ks_ was scaled to 20%, as done in our previous studies.^1,2^ The ionic properties of the different tissue regions were initialized to steady state by pacing at a basic cycle length of 450 ms.

#
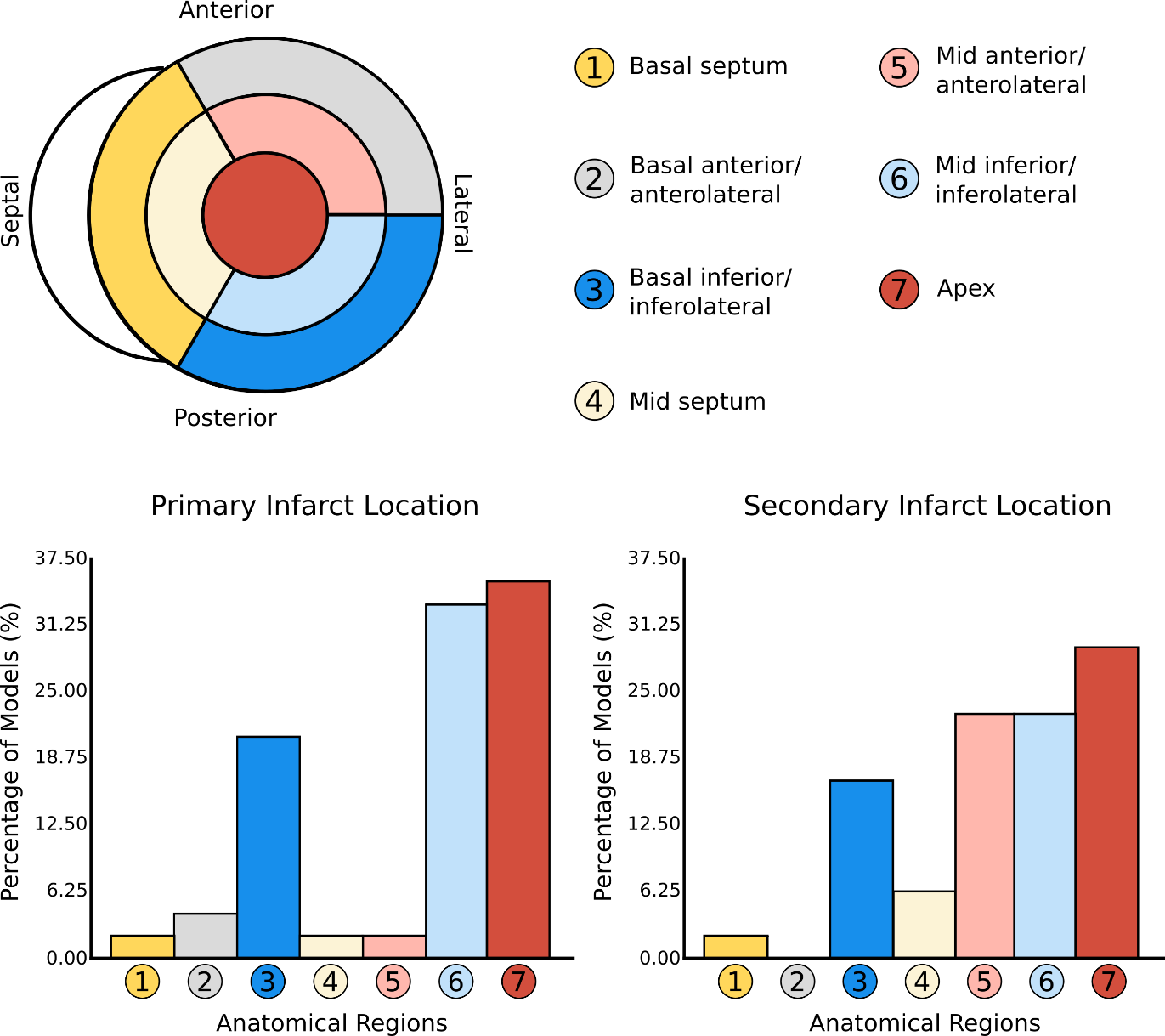
Supplemental Figures

**Supplemental Figure 1: Infarct distribution across heart models.** The top left panel is a modified American Heart Association bullseye diagram to illustrate the different anatomical regions (shown top right). The two bottom plots show the primary infarct location (bottom left) where infarct tissue most localized and the secondary infarct location (bottom right) where infarct tissue second most commonly localized. Most infarcts localized to the apex, inferior, and inferolateral regions.


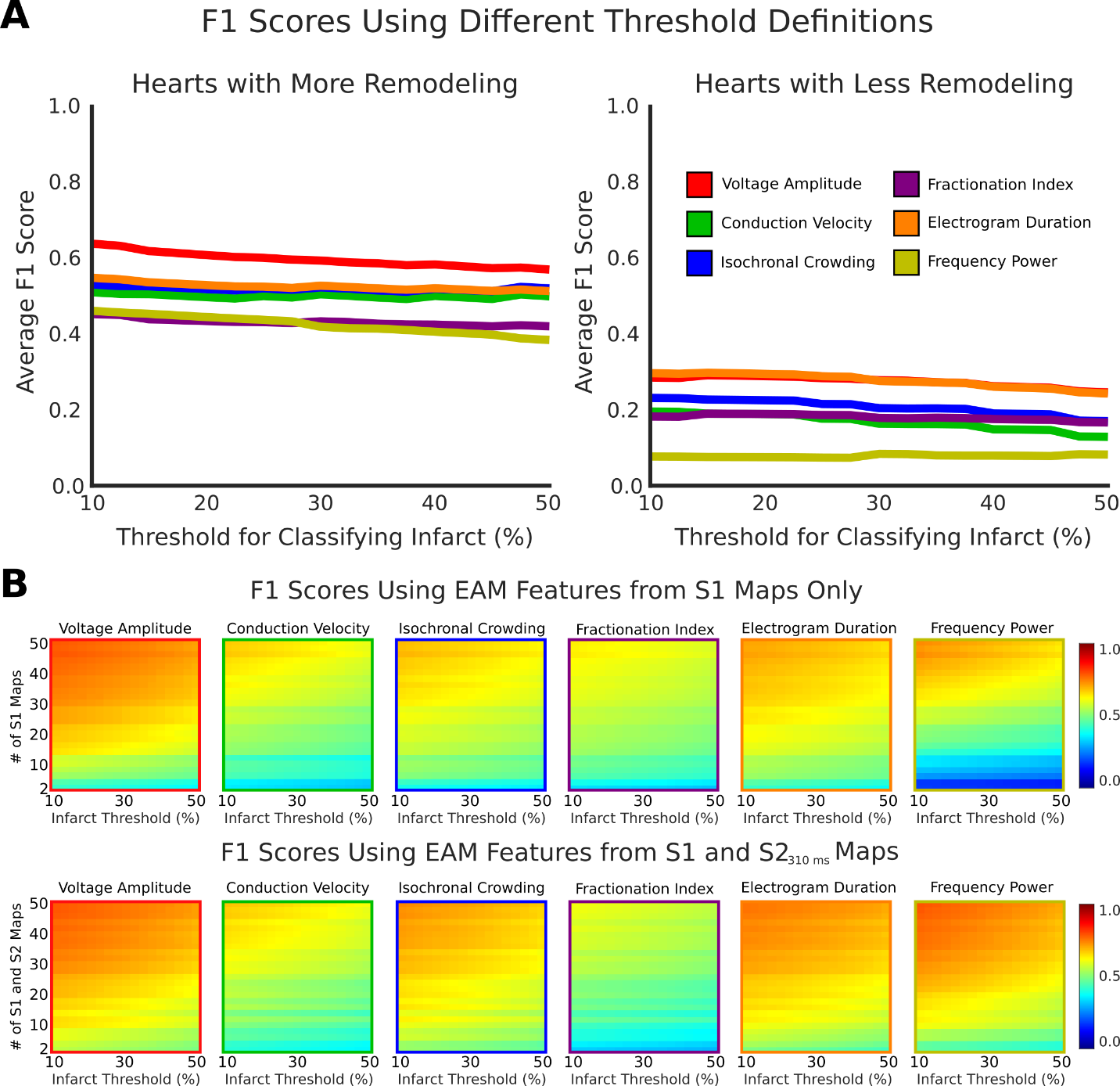


**Supplemental Figure 2:** **Assessment of how different infarct thresholds affects results.** The impact of varying the infarct threshold, the percentage threshold of tissue surrounding for classifying an EAM point as being in infarct, was assessed. **A:** Impact of infarct threshold on the predictive capabilities of EAM features from individual pacing sites. The plots show the F1 scores, averaged across all models and pacing sites, resulting from varying the threshold for classifying an EAM point as being in infarct versus not in infarct. Overall, there was a minor decrease in the ability of all EAM features to correctly identify the presence of infarct when the threshold for classifying an EAM point was increased from 10% to 50%. No matter the threshold, EAM features remained better at predicting the presence of infarct in hearts with more remodeling than in hearts with less remodeling. **B:** Impact of varying infarct threshold on the predictive capabilities of EAM features from multiple pacing sites. The panels show heat maps (F1 scores) of using EAM features from S1 maps alone (top) versus using EAM features from the equivalent number of both S1 and S2 maps (bottom). Even with a different percentage threshold for classifying infarct, a combination of EAM features from S1 and S2 maps remained superior to EAM features from S1 maps alone.

# References

1. Sung E, Prakosa A, Aronis KN, Zhou S, Zimmerman SL, Tandri H, *et al.* Personalized Digital-Heart Technology for Ventricular Tachycardia Ablation Targeting in Hearts with Infiltrating Adiposity. *Circulation: Arrhythmia and Electrophysiology* Lippincott Williams and Wilkins; 2020;

2. Prakosa A, Arevalo HJ, Deng D, Boyle PM, Nikolov PP, Ashikaga H, *et al.* Personalized virtual-heart technology for guiding the ablation of infarct-related ventricular tachycardia. *Nature Biomedical Engineering* Nature Publishing Group; 2018;**2**:732–40.
